# System Approach for Building of Calcium-Binding Sites in Proteins

**DOI:** 10.3390/biom10040588

**Published:** 2020-04-11

**Authors:** Alexander I. Denesyuk, Sergei E. Permyakov, Mark S. Johnson, Konstantin Denessiouk, Eugene A. Permyakov

**Affiliations:** 1 Institute for Biological Instrumentation of the Russian Academy of Sciences, Federal Research Center “Pushchino Scientific Center for Biological Research of the Russian Academy of Sciences”, Pushchino 142290, Russia; permyakov.s@gmail.com (S.E.P.); epermyak@yandex.ru (E.A.P.); 2 Structural Bioinformatics Laboratory, Faculty of Science and Engineering, Biochemistry, Åbo Akademi University, Turku 20520, Finland; johnson4@abo.fi (M.S.J.); kdenessi@abo.fi (K.D.); 3 Pharmaceutical Sciences Laboratory, Faculty of Science and Engineering, Pharmacy, Åbo Akademi University, Turku 20520, Finland

**Keywords:** system approach, protein structure, cation, calcium, sequence-structure motifs, building kit

## Abstract

We introduce five new local metal cation (first of all, Ca^2+^) recognition units in proteins: Clamp_n,(n−2)_, Clamp_n,(n−1)_, Clamp_n,n_, Clamp_n,(n+1)_ and Clamp_n,(n+2)_. In these units, the backbone oxygen atom of a residue in position “n” of an amino acid sequence and side-chain oxygen atom of a residue in position “n + i” (i = −2 to +2) directly interact with a metal cation. An analysis of the known “Ca^2+^-bound niches” in proteins has shown that a system approach based on the simultaneous use of the Clamp units and earlier proposed One-Residue (OR)/Three-Residue (TR) units significantly improves the results of constructing metal cation-binding sites in proteins.

## 1. Introduction

Many monographs, including ours [1,2], have been written about metal-binding proteins and metal cations. However, there are still too many uncertainties regarding how their interactions are organized. Biologically significant metals are divided into two groups: non-transition elements (Na, K, Mg, Ca, Zn) and transition elements (Mn, Fe, Co, Cu, Mo, W). Non-transition elements are characterized by the constancy of their oxidation state (valency) and formation of ions with incompletely filled s-electron shells or completely filled p-electron shells. In contrast, transition elements are characterized by variable valency (oxidation state) and the formation of ions with incompletely filled d-electron shells. Calcium ions, as well as magnesium, Na^+^ and K^+^ ions, are coordinated mainly by negatively charged oxygen atoms (see [1,2,3] for reviews). This is the case also for such heavy metals like Sr and Ba. The interaction is purely electrostatic. Ca^2+^ ions prefer a higher coordination number compared with Mg^2+^ ions. The usual coordination number for magnesium is six (octahedral coordination). Calcium demonstrates a lot more variety of coordination numbers, seven to nine being the most ordinary coordination numbers. The radius of the coordination sphere for calcium is essentially larger than that for magnesium: the distance from the central ion to oxygen atom is 2.0 to 2.1 Å for magnesium and 2.3 to 2.6 Å for calcium.

Ca^2+^ is a “hard” metal ion and prefers “hard” ligands with low polarizability, oxygen being the most preferable coordinating atom followed by nitrogen (reviewed by Dudev and Lim, 2003). Mg^2+^, like Ca^2+^, is also a “hard” ion and prefers “hard” oxygen-containing ligands as well. In contrast to Ca^2+^ and Mg^2+^ ions, Zn^2+^ ion and transition metal ions prefer “soft” polarizable ligands such as S and N though they are coordinated also by oxygen atoms. Here we will discuss only the binding sites for cations of non-transition metals.

**Figure 1:**
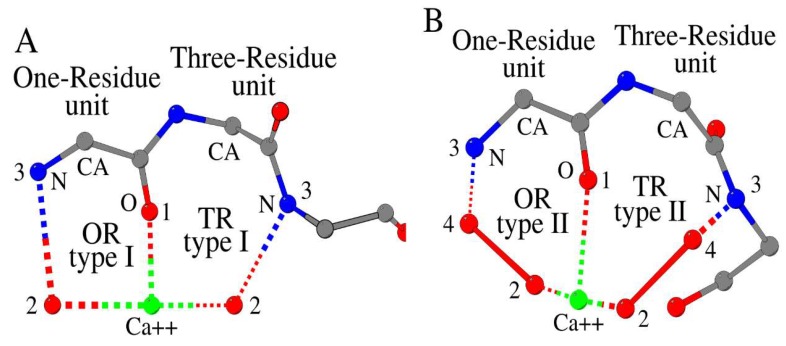
Metal cation-binding One-Residue (OR) and Three-Residue (TR) units, type I (**A**) and type II (**B**), in proteins. The difference between recognition by type I and type II is that the bound Ca^2+^ atom is linked to the main-chain nitrogen atom “3” through one oxygen atom (“2”, type I) or two oxygens atoms (“2” and “4”, type II). The line between atoms “2” and “4” is not a covalent bond, but a rigid connection between two atoms of the same amino acid or a ligand, or two adjacent amino acids (n) and (n − 1)/(n)/(n + 1). Amino acid atoms, water molecules and ligand atoms (carbon as gray, nitrogen as blue and oxygen as red) and cations as green are shown using the ball-and-stick model.

Recently, we found that the structure of a metal cation-binding site in proteins can be modeled using a set of four recognition units: One-Residue (OR) units of types I and II, and Three-Residue (TR) units of types I and II (Figure 1A,B, respectively). The universal key component of all four units is the main-chain oxygen (Position X), which directly interacts with cation. We named this set as a “Building Kit” [4].

**Figure 2:**
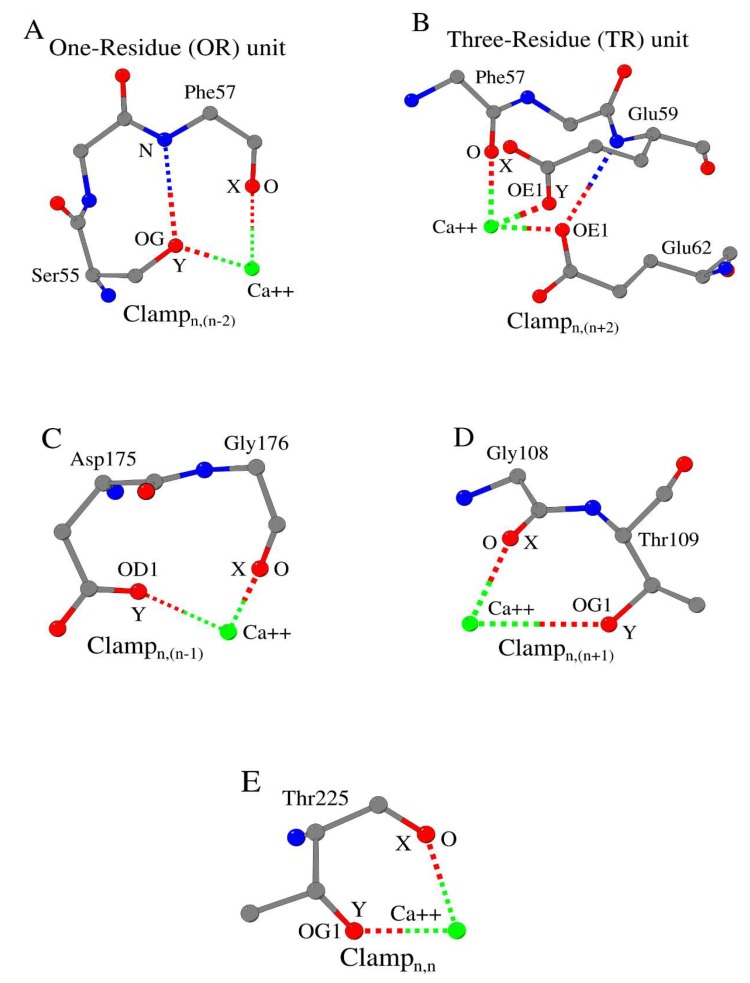
Five different types of local metal ion recognition substructures, observed in proteins: (**A**) Clamp_n,(n−2)_, (**B**) Clamp_n,(n+2)_, (**C**) Clamp_n,(n−1)_, (**D**) Clamp_n,(n+1)_ and (**E**) Clamp_n,n_. Main-chain and side-chain oxygen atoms, which directly coordinate the metal cation, are shown as “X” and “Y”.

In the formation of the ORI/II and TRI/II units, the participation of the side-chain groups of amino acids of the tripeptide (for example, fragment Phe57-Glu59 of pike parvalbumin pI 4.10 in Figure 2A,B), in which the first amino acid includes the above-mentioned main-chain oxygen, is not obligatory. However, the side-chain oxygen atom OE1 of Glu59 (Position Y) directly interacts with calcium in pike parvalbumin (Figure 2B) [5]. The possible exclusion of the atoms of the side-chain groups of the tripeptide in the construction of ORI/II and TRI/II units can partially explain the fact that the building kit, on average, includes only 70% of the atoms that coordinate bound metal cation [4].

On the other hand, we have also shown that the side-chain atom OG (Position Y) of amino acid Ser55 in pike parvalbumin, which is located symmetrically with respect to amino acid Glu59 relative to amino acid Phe57 in the pentapeptide, participates in the formation of the ORI unit (Figure 2A) [4]. This is due to the fact that the fragment Ser55-Phe57 forms the widespread secondary structure D/N/S/T-turn [6,7,8].

Here, we have analyzed the role of the side-chain groups of a pentapeptide, in which the main-chain oxygen of the central amino acid directly binds the cation, in the formation of both ORI/II, TRI/II units and new local cation-binding structures.

## 2. Materials and Methods

This publication is a continuation of our work on the identification of four local Ca^2+^-recognition units in proteins named ORI/II and TRI/II [4]. Previous analysis of metal cation-binding sites was carried out using 20 non-redundant structures with metal-bound functional “niches” [9] and 386 representative X-ray structures (≤30% sequence identity; resolution ≤1.50 Å) with bound Ca^2+^ atoms taken from the Protein Data Bank (PDB) [10,11]. The 20 “targeted” structures satisfied the following criterion: they contained a metal cation, which was bound to a main-chain carbonyl oxygen atom. A detailed justification for the selection of such a set of proteins was given in publication [4]. As a result, 25 PDB files were presented in the publication as three-dimensional structures containing various variants of the ORI/II and TRI/II units. Now we have analyzed the metal cation-binding sites in these 25 proteins and additionally in two Ca^2+^-binding proteins subtilisin Nat (PDB ID 3VYV) and annexin V (PDB ID 2IE7). We added these two proteins to the analysis since the structural organization of their Ca^2+^-binding sites is different from that of the Ca^2+^-binding sites in the homologous proteins subtilisin Carlsberg (PDB ID 1R0R) and annexin III (PDB ID 1AXN) in the 25-membered set.

Structure visualization and structural analysis of interactions between metal cations and the surrounding protein atoms was carried out using the Discovery Studio Modeling Environment (Dassault Systèmes BIOVIA, Discovery Studio Modeling Environment, Release 2017, San Diego: Dassault Systèmes, 2016) and the Ligand-Protein Contacts (LPC) software [12]. Color figures were produced with MOLSCRIPT [13].

## 3. Results and Discussion

### 3.1. Metal Cation-Binding Clamp_n,(n−2)_ Unit

We have analyzed the structures of calcium-binding sites in 27 proteins. It turned out that any calcium-binding site can be represented by a combination of separate elementary structural units. We called these units “Clamps”. The Clamp_n,(n−2)_ structural unit for metal binding can be described in terms of atoms that directly interact with a metal cation (Figure 2A). For example, in pike parvalbumin pI 4.10 (PDB ID 2PVB_A, Resolution (R) = 0.91 Å) [5]) the backbone oxygen atom of Phe57_n_ (Position X) and side-chain oxygen atom of Ser55_n−2_ (Position Y) directly interact with calcium (Table 1, Columns 5 and 6). Side-chain OG hydroxyl group of Ser55 also plays the role of an “atom-mediator” or bridging atom for the main-chain nitrogen of Phe57 and calcium [5]. As a result, the ORI_1_ “basic” unit is formed (Figure 1A, Table 1, Column 7 [4]). We used the term “basic” to emphasize the identity of the backbone oxygen that directly interacts with a metal cation in the formation of both Clamp_n,(n−2)_ and ORI_1_ units. The side-chain oxygen atom of Ser55 does not take part in the formation of any other OR or TR “extra” units (Table 1, Not Applicable or Not Appropriate (N/A) in Column 8). We used the term “extra” to emphasize that in the formation of any other OR or TR units, main-chain oxygen other than the main-chain oxygen of Phe57 must be used. Thus, Table 1 contains data not only about the atoms involved in the formation of the Clamp_n,(n−2)_ structural unit, but also data on the atoms involved in the formation of ORI/II and TRI/II units (Table 1, Columns 7 and 8 [4]). For example, a consideration of the location of Phe57 in pike parvalbumin pI 4.10 shows that it has both Clamp_n,(n−2)_ and ORI1 structural units in its calcium-binding site.

We found 12 metal cation (Ca, Na, K, Cs, Mg and Mn) binding sites that use the Clamp_n,(n−2)_ unit (Table 1, Columns 5 and 6). Eleven of them, as, for example, the sites in pike parvalbumin pI 4.10, simultaneously form the ORI_1_ unit. Only the calcium-binding site of stromelysin [14] possesses the ORII_1_ unit (Figure 1B). A structural description of the ORII_1_ unit was given in the work of Denesyuk et al. [4]. In stromelysin, in addition to participation in the formation of the ORII_1_ basic unit, the OD1/D182 atom is used also as a component of the TRII_1_ extra unit, in which Asp182 donates main-chain oxygen for direct binding of calcium (Figure 1B). The same structural pattern was found also in branched-chain α-ketoacid dehydrogenase, ligand K501 [15]. In ribokinase [16], the OD1/D249 atom participates in three extra units.

### 3.2. Metal Cation-Binding Clamp_n,(n+2)_ Unit

As we have noted above, pike parvalbumin pI 4.10 also has the Clamp_n,(n+2)_ structural unit, that is in this protein the backbone oxygen atom of Phe57_n_ (Position X) and side-chain oxygen atom of Glu59_n+2_ (Position Y) directly interact with calcium (Figure 2B and Table 2). However, the OE1/E59 atom does not participate in the formation of the TRI/II basic unit (Figure 1, Table 2, N/A in Column 7). Instead of the OE1/E59 atom, the OE1/E62 atom takes part in the formation of the TRI/II basic unit. Besides, the OE1/E59 atom does not participate in the formation of any other extra ORI/II or TRI/II units (Table 2, N/A in Column 8). Therefore, in this case (lack of the basic/extra ORI/II or TRI/II units: N/A-N/A in Columns 7 and 8 of Table 2), the inclusion of the Clamp_n,(n+2)_ unit in the building kit increases the number of modeled atoms that coordinate the bound metal cation. In order to mark the Clamp units possessing such property, we marked the atom in Position Y in bold (Table 2, Column 6).

There are four possible variants of the participation of an atom in the Position Y of the Clamp_n,(n+2)_ unit in the formation of basic and extra units: (1) N/A − N/A, (2) N/A − OR/TR, (3) OR/TR − N/A and (4) OR/TR − OR/TR. Variants 1 and 2 are the most common variants. In serralysin, Variant 2 repeats six times. Variant 4 is totally absent in the analyzed structures. For the Clamp_n,(n−2)_ unit, Variant 3 is the most characteristic (Table 1).

In our systematic analysis of the calcium-binding sites containing the niche motif, we also observed Variant 1 of the Clamp_n,(n−2)_ unit for trypsin: PDB ID 4I8H_A, Ca301_A; Position X, O/N72; Position Y, OE1/E70 [29]. Potentially, this is explained by the presence of a long amino acid Glu in position (n − 2) instead of a short one (Asp, Asn, Ser and Thr).

### 3.3. Metal Cation-Binding Clamp_n,(n−1)_ and Clamp_n,(n+1)_ Units

We found only four examples of the Clamp_n,(n−1)_ unit (Figure 2C, Table 3). In all four proteins, the atom in Position Y does not participate in the formation of the basic unit. Three proteins demonstrate a structurally homologous Variant 2 in the formation of the basic and extra units: (N/A − OR/TR). Sodium-binding site of dialkylglycine decarboxylase shows that Clamp_n,(n−1)_, as Clamp_n,(n−2)_ and Clamp_n,(n+2)_ units, must be included in the building kit for cation-binding sites.

Clamp_n,(n+1)_ unit is the rarest Clamp unit in comparison with other Clamp units involved in the formation of cation-binding sites (Figure 2D, Table 3). Both Clamp_n,(n−1)_ and Clamp_n,(n+1)_ units are equally significant for the formation of the basic and extra units.

### 3.4. Metal Cation-Binding Clamp_n,n_ Unit

Table 4 shows 11 examples of the use of the Clamp_n,n_ unit (Figure 2E) in the calcium-binding sites of proteins. Unlike the four previous types of Clamp units, we found all possible variants of the Clamp_n,n_ unit participation in the formation of the basic and extra units.

### 3.5. System Approach in a Joint Use of OR/TR and Clamp Units

Five new local units, Clamp_n,(n−2)_, Clamp_n,(n−1)_, Clamp_n,n_, Clamp_n,(n+1)_ and Clamp_n,(n+2)_ have been revealed in spatial structures of the metal cation-binding sites of proteins. Side-chain oxygens of these Clamp units are involved in the formation of both basic and extra OR/TR units. The combination of OR/TR and Clamp units in the same building kit makes it possible to increase the number of modeled atoms that coordinate bound metal cation.

**Figure 3:**
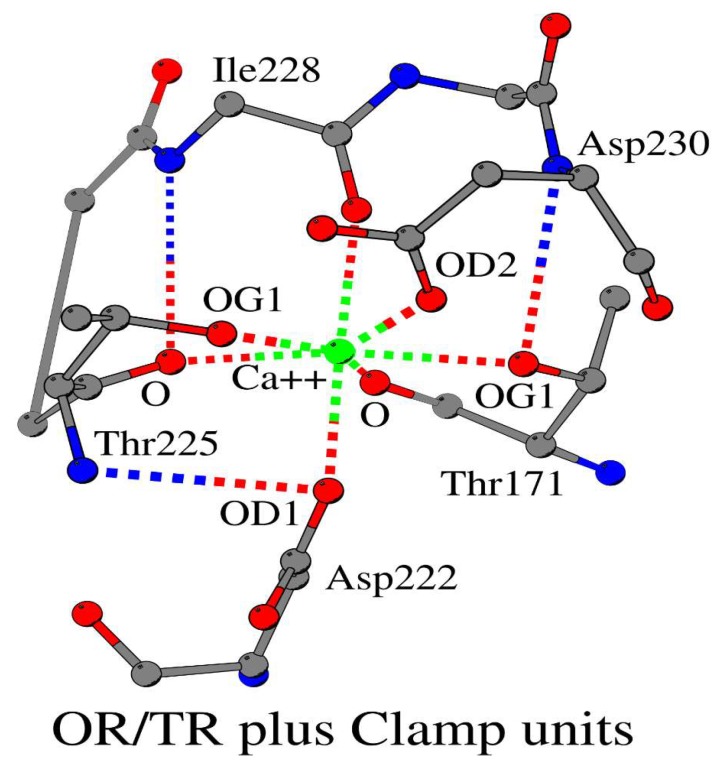
Atomic cation-binding network in peroxidase. Three OR/TR and three Clamp units are shown.

Let us show how the OR/TR and Clamp units are used by Nature to design the complete structure of a calcium (Ca1308_A)-binding site using a three-dimensional structure of peroxidase (PDB ID 1GWU_A) as an example. The mutual spatial arrangement of the OR/TR and Clamp units in this metal cation-binding site is shown in Figure 3. It has three OR/TR units: O/Thr225 (ORI_1_) and O/Ile228 (ORI_2_ and TRI_1_). Their structures include four metal cation-binding atoms: OD1/Asp222, O/Thr225, O/Ile228 and OG1/Thr171. ORI_2_ plus TRI_1_ units form ADA–DAD (Acceptor/Donor/Acceptor–Donor/Acceptor/Donor) structural motif, which we described in detail earlier [5,33]. There are also three types of the Clamp units in this metal cation-binding site: O/Thr171 (Clamp_n,n_), O/Thr225 (Clamp_n,n_) and O/Ile228 (Clamp_n,(n+2)_). Their structures include six cation-binding atoms: O/Thr171, OG1/Thr171, O/Thr225, OG1/Thr225, O/Ile228 and OD2/D230. A combination of these two sets of atoms provides all seven cation-binding atoms: O/Thr171, OG1/Thr171, OD1/Asp222, O/Thr225, OG1/Thr225, O/Ile228 and OD2/D230. The use of seven instead of four atoms in the modeling of the cation-binding site clearly shows the benefits of using the Clamp units. Three atoms OG1/Thr171, O/Thr225 and O/Ile228 are parts of both the OR/TR and Clamp units. The atom OG1/Thr171 and atom O/Thr225 are structural “twins” with respect to the calcium and the O/Ile228 atom. Undoubtedly, the simultaneous use of the nitrogen and oxygen of the main-chain atoms, as well as the oxygen of the side-chain groups of small fragments of the amino acid sequence of the protein to form a cation-binding site is an evolutionarily selected result.

### 3.6. Hierarchy of OR/TR and Clamp units

Water molecules and some other ligands in the Ca^2+^-binding site do not participate directly in the formation of the Clamp units. At the same time, they can participate in the formation of the ORI/II and TRI/II units [4]. The pentapeptide that has metal-binding oxygen in its middle and forms ORI/II and TRI/II units, in some cases, may contain T/S/D/N/E/Q amino acids. It can be assumed that the presence of such ligands in the Ca^2+^-binding site prevents the formation of some Clamp units. Potentially, these T/S/D/N/E/Q amino acids fulfill some other more important roles and not just participation in the formation of the Ca^2+^-binding site. In this case, the appearance of water molecules and some other ligands in the Ca^2+^-binding site helps to compensate for the absence of the Clamp units. This implies the hierarchy in the use of the structural units in constructing of the Ca^2+^-binding sites: OR/TR units are used first and then Clamp. One of the possible structural explanations for this hierarchy is that only main-chain atoms of the tripeptide participate in ORI/II and TRI/II units, and some atoms of pentapeptide in Clamp units are side-chain atoms.

## 4. Conclusions

In the present study, we determined five new local metal cation recognition units in proteins: Clamp_n,(n−2)_, Clamp_n,(n−1)_, Clamp_n,n_, Clamp_n,(n+1)_ and Clamp_n,(n+2)_. Since the interactions of the cations of non-transition elements with their ligands are purely electrostatic without any selected directions, one could suggest that their binding sites in proteins should have a simple design. The results of our work show that this is not the case: these binding sites and their surroundings have a rather complex structure. Nevertheless, they can be presented as a sum of evolutionary selected simple elements, metal cations recognition elements, revealed in our studies. The elements of this “building kit” can be used in protein engineering for the design of metal-binding sites in proteins.

It should be noted that the use of the OR/TR and Clamp structural units cannot explain the construction of absolutely all Ca^2+^-binding sites. The units found in the present work are based on the binding sites, which contain the main-chain carbonyl oxygen taking part in the coordination of metal ions. There are Ca^2+^-binding sites, which do not contain main-chain carbonyl oxygens. Moreover, the total structure of some metal-binding sites containing the main-chain carbonyl oxygen cannot be explained using only the OR/TR and Clamp units. Our experience shows that quite often one can explain the tertiary arrangement of three or four out of six possible chelators of a metal atom. The goal of our two publications, the present one and the previous one [4], is to lay the foundation for the creation of a complete “full-fledged building kit”.

## Figures and Tables

**Table 1:** Existence of the Clamp_n,(n−2)_ unit in the metal cation-binding sites of proteins with “niche” motifs in these sites.

N	Protein	PDB ID, R (Å)	Ligand	Atom, p. X	Atom, p. Y	Basic Unit	Extra Unit	Ref.
0	Parvalbumin	2PVB_A, 0.91	Ca110_A	O/F57	OG/S55	ORI_1_	N/A	[5]
1	BCKD (branched-chain α-ketoacid DH)	2BFD_A, 1.39	K501_A	O/P163	OG/S161	ORI_1_	S161, TRI_1_	[15]
2	BCKD (branched-chain α-ketoacid DH)	2BFD_A, 1.39	Mn503_A	O/Y224	OD1/N222	ORI_1_	N/A	[15]
3	Factor Xa	2Y5F_A, 1.29	Na1245_A	O/N72	OD1/D70	ORI_1_	N/A	[17]
4	Pyruvate dehydrogenase	2OZL_A, 1.90	Mg2331_A	O/Y198	OD1/N196	ORI_1_	N/A	[18]
5	Ribokinase	1GQT_A, 2.34	Cs1309_A	O/I251	OD1/D249	ORI_1_	D249, TRI_1_, R288, TRII_11_, G290, ORII_11_	[16]
6	Serralysin	5D7W_A, 1.10	Ca502_A	O/G287	OD1/D285	ORI_1_	N/A	[19]
7	Sphericase	2IXT_A, 0.80	Ca1311_A	O/V219	OD2/D217	ORI_1_	N/A	[20]
8	Stromelysin	1HY7_A, 1.50	Ca304_A	O/E184	OD1/D182	ORII_1_	D182, TRII_1_	[14]
9	Subtilisin Carlsberg	1R0R_E, 1.10	Ca302_E	O/T79	OD1/N77	ORI_1_	N/A	[21]
10	Subtilisin Nat	3VYV_A, 1.36	Ca303_A	O/I79	OD1/N77	ORI_1_	N/A	[22]
11	Thermitase	1THM_A, 1.37	Ca301_A	O/T87	OD1/N85	ORI_1_	N/A	[23]
12	Thermitase	1THM_A, 1.37	Ca302_A	O/T64	OD1/D62	ORI_1_	N/A	[23]

**Table 2:** Existence of the Clamp_n,(n+2)_ unit in the metal cation-binding sites of proteins with “niche” motifs in these sites.

N	Protein	PDB ID, R (Å)	Ligand	Atom, p. X	Atom, p. Y	Basic Unit	Extra Unit	Ref.
0	Parvalbumin	2PVB_A, 0.91	Ca110_A	O/F57	**OE1/E59**	N/A	N/A	[5]
1	Annexin III	1AXN_A, 1.78	Ca355_A	O/T193	OE1/E195	TRI_1_	N/A	[24]
2	Annexin V	2IE7_A, 1.75	Ca407_A	O/T31	OE1/E33	TRI_1_	N/A	[25]
3	Calcium pump	1SU4_A, 2.40	Ca995_A	O/I307	OE1/E309	TRI_1_	N/A	[26]
4	Dialkylglycine decarboxylase	1M0Q_A, 2.00	K434_A	O/L78	**OG/S80**	N/A	N/A	[27]
5	Dialkylglycine decarboxylase	1M0Q_A, 2.00	K434_A	O/V305	OD1/D307	N/A	L78, ORI_1_	[27]
6	Peroxidase	1GWU_A, 1.31	Ca1307_A	O/G48	**OD1/D50**	N/A	N/A	[28]
7	Peroxidase	1GWU_A, 1.31	Ca1308_A	O/I228	**OD2/D230**	N/A	N/A	[28]
8	Serralysin	5D7W_A, 1.10	Ca502_A	O/G255	OG1/T257	N/A	G287, TRII_4_	[19]
9	Serralysin	5D7W_A, 1.10	Ca503_A	O/G288	OD2/D290	N/A	T327, TRII_9_	[19]
10	Serralysin	5D7W_A, 1.10	Ca503_A	O/T327	OE2/E329	N/A	G228, TRI_1_	[19]
11	Serralysin	5D7W_A, 1.10	Ca504_A	O/G336	OD2/D338	N/A	A353, TRII_4_	[19]
12	Serralysin	5D7W_A, 1.10	Ca505_A	O/A345	OD1/N347	N/A	G362, TRII_4_	[19]
13	Serralysin	5D7W_A, 1.10	Ca506_A	O/G354	OD2/D356	N/A	A371, TRII_4_	[19]
14	Serralysin	5D7W_A, 1.10	Ca507_A	O/G372	**OD2/D374**	N/A	N/A	[19]
15	Serralysin	5D7W_A, 1.10	Ca508_A	O/G363	**OD2/D365**	N/A	N/A	[19]
16	Sphericase	2IXT_A, 0.80	Ca1310_A	O/G297	OD1/D299	N/A	A295, ORI_1_	[20]
17	Sphericase	2IXT_A, 0.80	Ca1311_A	O/V219	**OE1/Q221**	N/A	N/A	[20]
18	Stromelysin	1HY7_A, 1.50	Ca305_A	O/N175	OD1/D177	N/A	D141, TRI_1_	[14]
19	Subtilisin Carlsberg	1R0R_E, 1.10	Ca302_E	O/L75	OD1/N77	N/A	T79, ORI_1_	[21]
20	Subtilisin Nat	3VYV_A, 1.36	Ca303_A	O/L75	OD1/N77	N/A	I79, ORI_1_	[22]
21	Subtilisin Nat	3VYV_A, 1.36	Ca304_A	O/E195	OD2/D197	N/A	T174, TRI_1_	[22]
22	Thermitase	1THM_A, 1.37	Ca302_A	O/T64	OE1/Q66	TRI_1_	N/A	[23]

Those atoms that do not participate in the formation of basic and extra ORI/II or TRI/II units are marked in bold.

**Table 3:** Existence of the Clamp_n,(n−1)_ and Clamp_n,(n+1)_ units in the metal cation-binding sites of proteins with “niche” motifs in these sites.

N	Protein	PDB ID, R (Å)	Ligand	Atom, p. X	Atom, p. Y	Basic Unit	Extra Unit	Ref.
Clamp_n,(n−1)_ unit								
1	Dialkylglycine decarboxylase	1M0Q_A, 2.00	Na436_A	O/P99	**OG1/T98**	N/A	N/A	[27]
2	Fibroblast collagenase	1HFC_A, 1.50	Ca277_A	O/G176	OD1/D175	N/A	G178, TRII_11_, N180, ORII_11_	[30]
3	Sphericase	2IXT_A, 0.80	Ca1310_A	O/I288	OD1/D287	N/A	A295, TRII_11_, G297, ORII_11_	[20]
4	Stromelysin	1HY7_A, 1.50	Ca303_A	O/G159	OD1/D158	N/A	G161, TRII_11_, V163, ORII_11_	[14]
	Clamp_n,(n+1)_ unit							
1	Annexin III	1AXN_A, 1.78	Ca352_A	O/G108	**OG1/T109**	N/A	N/A	[24]
2	BCKD (branched-chain α-ketoacid DH)	2BFD_B, 1.39	K502_B	O/L130	OG1/T131	N/A	N183, TRII_1_	[15]

Those atoms that do not participate in the formation of basic and extra ORI/II or TRI/II units are marked in bold.

**Table 4:** Existence of the Clamp_n,n_ unit in the metal cation-binding sites of proteins with “niche” motifs in these sites.

N	Protein	PDB ID, R (Å)	Ligand	Atom, p. X	Atom, p. Y	Basic Unit	Extra Unit	Ref.
1	Annexin V	2IE7_A, 1.75	Ca403_A	O/D224	OD1/D224	N/A	T227, ORII_1_	[25]
2	BCKD (branched-chain α-ketoacid DH)	2BFD_A, 1.39	K501_A	O/S161	OG/S161	TRI_1_	P163, ORI_1_	[15]
3	Dialkylglycine decarboxylase	1M0Q_A, 2.00	Na436_A	O/T98	**OG1/T98**	N/A	N/A	[27]
4	Homoserine dehydrogenase	1EBF_A, 2.30	Na2104_A	O/E143	OE2/E143	TRII_1_	L150, TRI_1_	[31]
5	NaCl-dependent neurotransmitter transporter	2A65_A, 1.65	Na752_A	O/T254	OG1/T254	TRII_1_	N/A	[32]
6	Peroxidase	1GWU_A, 1.31	Ca1307_A	O/D43	OD1/D43	N/A	G48, TRI_1_	[28]
7	Peroxidase	1GWU_A, 1.31	Ca1308_A	O/T171	OG1/T171	N/A	I228, TRI_1_	[28]
8	Peroxidase	1GWU_A, 1.31	Ca1308_A	O/T225	**OG1/T225**	N/A	N/A	[28]
9	Ribokinase	1GQT_A, 2.34	Cs1309_A	O/D249	OD1/D249	TRI_1_	I251, ORI_1_, R288, TRII_11_, G290, ORII_11_	[16]
10	Stromelysin	1HY7_A, 1.50	Ca304_A	O/D182	OD1/D182	TRII_1_	E184, ORII_1_	[14]
11	Subtilisin Nat	3VYV_A, 1.36	Ca304_A	O/T174	**OG1/T174**	N/A	N/A	[22]

Those atoms that do not participate in the formation of basic and extra ORI/II or TRI/II units are marked in bold.
